# Highly Sensitive Carbon Monoxide Sensor Element with Wide-Range Humidity Resistance by Loading Pd Nanoparticles on SnO_2_ Surface

**DOI:** 10.3390/s22082934

**Published:** 2022-04-11

**Authors:** Koichi Suematsu, Akihito Uchiyama, Ken Watanabe, Kengo Shimanoe

**Affiliations:** 1 Department of Advanced Materials Science and Engineering, Faculty of Engineering Sciences, Kyushu University, Kasuga, Fukuoka 816-8580, Japan; suematsu.koichi.682@m.kyushu-u.ac.jp (K.S.); watanabe.ken.331@m.kyushu-u.ac.jp (K.W.); 2 Department of Molecular and Material Science, Interdisciplinary Graduate School of Engineering Science, Kyushu University, Kasuga, Fukuoka 816-8580, Japan; uchiyama.akihito.698@s.kyushu-u.ac.jp

**Keywords:** SnO_2_, Pd loading, colloidal protection method, CO sensor, humidity resistance

## Abstract

To develop a highly sensitive carbon monoxide (CO) sensor with a wide range of humidity resistance, we focused on the Pd loading method on SnO_2_ nanoparticles and the thickness of the sensing layer. The Pd nanoparticles were loaded on the SnO_2_ surface using the surface immobilization method (SI-Pd/SnO_2_) and the colloidal protection method (CP-Pd/SnO_2_). The XPS analysis indicated that the Pd nanoparticles were a composite of PdO and Pd, regardless of the loading method. According to the evaluation of the electrical properties at 350 °C, the CO response in a humid atmosphere and the resistance toward humidity change using CP-Pd/SnO_2_ were higher than those using SI-Pd/SnO_2_, even though the Pd loading amount of SI-Pd/SnO_2_ was slightly larger than that of CP-Pd/SnO_2_. In addition, Pd/SnO_2_ prepared via the CP method with a thinner sensing layer showed a higher sensor response and greater stability to humidity changes at 300 °C, even though the humidity change influenced the CO response at 250 and 350 °C. Thus, the overall design of the surface Pd, including size, dispersity, and oxidation state, and the sensor fabrication, that is, the thickness of the sensing layer, offer a high-performance semiconductor-type CO gas sensor with a wide range of humidity resistance.

## 1. Introduction

Carbon monoxide (CO) is widely emitted in the domestic and industrial fields, for example, in heating systems and vehicle and power plant emissions, even though it is a harmful gas because of its toxic activity. As a critical issue, poor combustion of the gas heating system produces CO, which often causes CO poisoning in humans. Thus, rapid and stable detection in the low-concentration range is in significant demand for CO-detection devices. As one candidate, the tin dioxide (SnO_2_)-based semiconductor gas sensor has been widely investigated because of its high sensitivity, long-term stability, and rapid detection ability [1,2,3,4,5,6,7]. However, the semiconductor gas sensor has a fundamental issue in that it degrades in the presence of humidity, and the sensor response is drastically influenced by humidity changes in the atmosphere [8,9,10,11,12,13]. From another viewpoint, it is possible to correct the sensor signal of the semiconductor gas sensor using the signal of a humidity sensor. Due to the recent development of micro-electro mechanical systems (MEMS), both semiconductor gas sensors and humidity sensors can be mounted in the small sensor device. However, it is not the best way to apply a compact and low power-consumption sensor device. Thus, the development of high-performance gas sensors with a wide range of resistance to changes in humidity and a high sensor response to CO is necessary.

Generally, the electrical resistance of SnO_2_-based semiconductor gas sensors is attributed to the quantity of the dissociative adsorbed oxygen, as O^−^ and O^2−^, on the particle surface [6,14,15,16,17,18]. When combustible gases such as CO are present in the atmosphere, the gas molecules react with the adsorbed oxygen, and the electrical resistance decreases. Such a change in electrical resistance signals is necessary for the detection of gas molecules. However, if humidity is present in the atmosphere, the hydroxyl groups cover the oxygen adsorption site, resulting in hydroxyl poisoning [6,12,19,20]. As a result, the electrical resistance and sensor response are reduced by the disturbed oxygen adsorption.

Many studies have been previously conducted on the prevention of hydroxyl poisoning [21,22,23,24,25,26,27]. Additive secondary elements, such as oxides and metal particles, are an effective way to restrict hydroxyl poisoning. For example, some additives, such as NiO [21], CuO [22], Al [23], and Sb [24], act as hydroxyl absorbers and prevent the formation of hydroxyl groups on the oxygen adsorption site. The addition of catalytic metal particles such as Pd and Pt [24,25,26,27] is also an attractive way to restrict hydroxyl poisoning. For example, we previously proposed that Pd on the SnO_2_ surface provides oxygen adsorption sites and protects against hydroxyl poisoning [27]. On the other hand, Marikutsa et al. reported two CO detection mechanisms on the Pd/SnO_2_ surface in a wet atmosphere [26]. One was that the oxidized Pd, PdO, on SnO_2_ surface preferentially reacts with CO. It reduces the PdO, and decreases the electrical resistance of Pd/SnO_2_, which is based on the conventional electronic sensitization model [28,29,30]. The other was that surface OH directly reacts with CO, and produced H^+^ reacts with adsorbed oxygen, which is similar to the water-gas-shift reaction between CO and H_2_O [31,32]. Hahn et al. proposed that CO reacts with hydroxyls on the surface of SnO_2_ to produce the CO_2_ and H adsorbed on the oxygen, and this H decreases the electrical resistance [33]. However, according to the report, the ratio of the water-gas-shift reaction on Pd is lower than other novel metal catalysts [31,32]. Nevertheless, in either case, Pd loading on SnO_2_ surface enhances the CO response in a wet atmosphere because of the activation of CO combustion. 

Pd/SnO_2_ is an excellent candidate for a highly sensitive semiconductor CO sensor with humidity resistance. Accordingly, Pd/SnO_2_ have been widely used for the commercial gas sensor [1,2,3], and have been widely investigated for the development of further high-performance sensing materials. Previously, we changed the characteristics of Pd by using different starting materials and investigated the sensor performance in a wet atmosphere. Accordingly, we reported that the particle size of Pd and the oxidized state are effective in providing humidity resistance to the sensor performance owing to the catalytic combustion of Pd [34,35]. In addition, we previously indicated that increasing the Pd loading amount over 1 mol% with a small particle size and high dispersity improves the sensor response to CO in a wet atmosphere [27]. Thus, in this study, we aimed to develop high-performance Pd/SnO_2_ nanoparticles, focusing on the Pd loading method with small size and high dispersity and the thickness of the sensing layer as an overall sensor element design. According to the results, we finally achieved a high sensor response to CO with high stability to changes in humidity by combining the selection of the Pd loading method and the fabrication of a thinner sensing layer at the optimum operating temperature. 

## 2. Materials and Methods

### 2.1. Synthesis of SnO_2_ and Pd/SnO_2_ Nanoparticles and Sensor Fabrication

**Figure 1:**
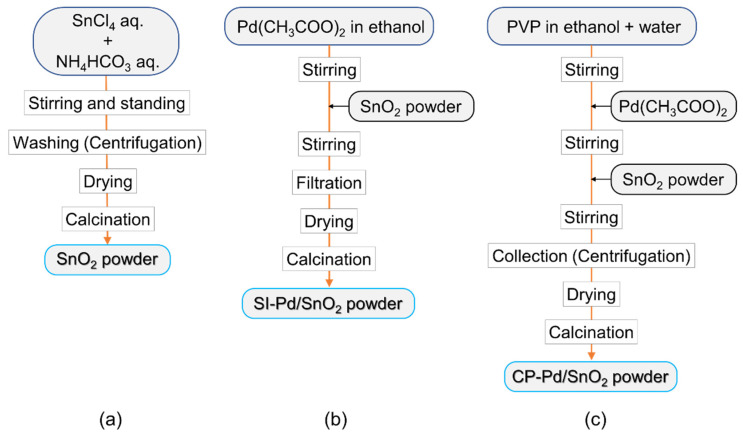
Flow chart of (**a**) the SnO_2_ preparation process, and Pd loading procedures based on the (**b**) surface immobilization and (**c**) colloidal protection methods.

Bare-SnO_2_ nanoparticles were synthesized starting from SnCl_4_·5H_2_O (FUJIFILM Wako Pure Chemical Corporation, Osaka, Japan). The SnCl_4_ solution (1 M) was placed in an NH_4_HCO_3_ (FUJIFILM Wako Pure Chemical Corporation, Osaka, Japan) solution in a drop-wise manner, and a white precipitate, a stannic acid gel, was obtained after 12 h. The obtained gel was dried at 120 °C for 12 h and calcined at 600 °C for 3 h to obtain SnO_2_ nanoparticles after washing by centrifugation using deionized water. Pd nanoparticles were loaded onto the SnO_2_ nanoparticles using two different processes. A flow chart of the SnO_2_ synthesis process and the two types of Pd loading processes are shown in Figure 1. Pd was loaded from Pd acetate (Pd(CH_3_COO)_2_, Tokyo Chemical Industry Co., Ltd., Tokyo, Japan) via two different procedures. One was based on the surface immobilization method (SI method), in which SnO_2_ powder was added to a solution of Pd acetate in ethanol with stirring. Pd/SnO_2_ powder was obtained by drying and calcining at 580 °C for 3 h after filtering the SnO_2_ powder with colloidal Pd particles. The obtained Pd/SnO_2_ is referred to as SI-Pd/SnO_2_. In another loading method, polyvinylpyrrolidone (PVP; FUJIFILM Wako Pure Chemical Corporation, Osaka, Japan) was dissolved in an ethanol and water mixture (the volume ratio of ethanol in the ethanol–water mixture was 70%) at 60 °C, and then Pd acetate and SnO_2_ powder were sequentially added to the solution. Colloidal PVP-Pd particles were produced by heating at 120 °C for 3 h. The obtained SnO_2_ with PVP-Pd colloidal particles was collected by centrifugation after cooling, drying, and calcining at 580 °C for 3 h to obtain Pd/SnO_2_ nanoparticles. In this process, colloidal Pd nanoparticles were protected by PVP [36]. Herein, Pd/SnO_2_ is referred to as CP-Pd/SnO_2_. The molar ratio of Pd was set to 3 mol% for each Pd loading method.

The sensing film was fabricated using the screen-printing technique on an alumina substrate (9 × 13 × 0.38 mm, Japan Fine Ceramics Company, Ltd., Miyagi, Japan) printed with a comb-type gold electrode (distance between the lines: 90 μm, area of the sensing layer: 64 mm^2^). The synthesized powders were mixed with α-terpineol (FUJIFILM Wako Pure Chemical Corporation, Osaka, Japan) to prepare the sensor paste, which was screen-printed on the substrate. The thickness of the sensing layer was controlled to approximately 14 and 30 μm by the ratio of the powders and α-terpineol. Here, ratios of the powder and α-terpineol were approximately 0.1 g to 240 μL and 0.1 g to 60 μL for thicknesses of 14 and 30 μm, respectively. The sensor element was attached to the electrical resistance measurement system with a gas mixture and then sintered at 550 °C to prepare the sensor element.

### 2.2. Materials and Sensor Evaluation

Pd loading amount on the SnO_2_ surface was evaluated using wavelength-dispersive X-ray fluorescence spectroscopy (WDX; Rigaku, ZSX Primus II, Yamanashi, Japan). The Pd loading amount was determined by the calibration curve method using Pd/SnO_2_ for calibration curves with known concentrations. SnO_2_ and Pd nanoparticles were observed by using a high-resolution transmission electron microscope (TEM; JEM-ARM200F, JEOL Ltd., Tokyo, Japan) at 200 kV. The crystal structures of the powders were evaluated by X-ray diffraction (XRD; Miniflex, Rigaku, Yamanashi, Japan) with Cu*Kα* radiation. Specific surface area and pore distribution were analyzed through nitrogen gas adsorption/desorption using a specific surface area and pore size distribution analyzer (BELSORP-mini II, MicrotracBEL, Osaka, Japan). The oxidation state of the Pd nanoparticles was evaluated using X-ray photoelectron spectroscopy (XPS; KRATOS ESCA 3400, Kyoto, Shimadzu). The binding energy was calibrated using the C1s peak (285.0 eV), and a Shirley-type background correction was applied for assignment.

**Figure 2:**
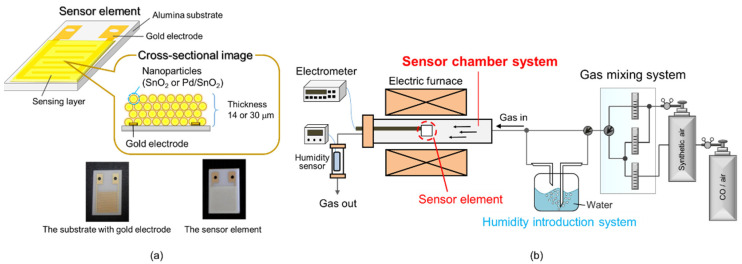
(**a**) Schematic image and photographs of the sensor element and (**b**) schematic image of measurement apparatus.

The electrical properties of the sensor element were measured using the apparatus equipped with a gas mixing system, humidity introduction systems with a volume of approximately 163 cm^3^, sensor measurement chamber with a volume of approximately 39.7 cm^3^, and a humidity sensor, as schematically described in Figure 2b. The humidity was introduced by blowing deionized water using the synthetic air gas, and humidity was controlled by mixing of dry and wet gases. The humidity of the measured gas was monitored using a capacitance-type commercial humidity gas sensor (TR-77Ui; T&D Corporation, Matsumoto, Japan). The total gas flow rate was adjusted to 100 cm^3^/min with mass flow controllers (SEC-series; HORIBA STEC, Kyoto, Japan). The sample gases containing CO and humidity in air were prepared by diluting the parent gas with purified synthetic air (air). The sensor element was connected to a standard resistance and voltage across the standard resistance was measured under an applied voltage (4 V) to estimate the electrical resistance of the sensor, and the electrical resistance of the sensor was acquired with an electrometer (Model 2701; Keithley Instruments, Ohio, USA). The sensor response was defined as *R_a_/R_g_*, where *R_a_* and *R_g_* are the electrical resistance in air and CO including air, respectively. 

## 3. Results and Discussion

### 3.1. Effect of Pd Loading Method on Materials and Electrical Characteristics

**Figure 3:**
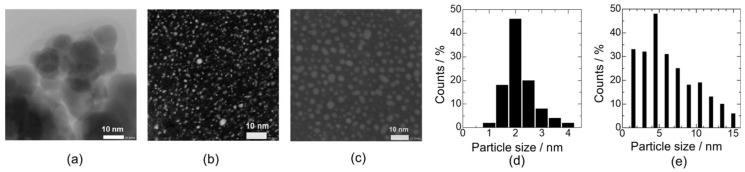
TEM images of (**a**) SnO_2_ nanoparticles, and Pd nanoparticles after the drying process using the (**b**) SI and (**c**) CP methods without SnO_2_ nanoparticles. Particle size distributions of the Pd nanoparticles using the (**d**) SI and (**e**) CP methods.

A TEM image of the bare-SnO_2_ nanoparticles is shown in Figure 3a, and the estimated particle size of SnO_2_ was approximately 15 nm. There was a difference in the Pd nanoparticles after the preparation of Pd using the SI and CP methods without SnO_2_ addition. Figure 3b,c show TEM images of dried Pd nanoparticles using the SI and CP methods, respectively, and the particle size distributions were estimated as shown in Figure 3d,e, respectively. According to the particle size distributions, the average particle sizes of SI-Pd and CP-Pd were 1.9 and 5.8 nm, respectively. This large difference in the average particle size was probably caused by the PVP protection layer on the Pd nanoparticles of CP-Pd. Thus, the Pd particle sizes in each loading process were clearly smaller than those of SnO_2_, and the Pd nanoparticles obtained via the CP method were successfully coated with PVP.

**Figure 4:**
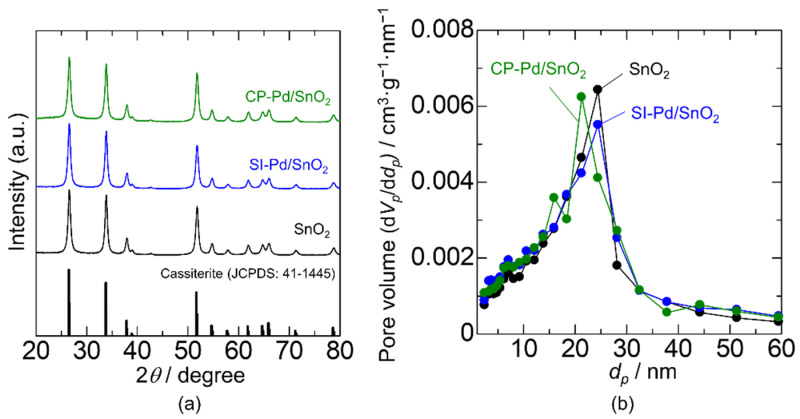
(**a**) XRD patterns and (**b**) pore distribution-based BJH estimation for SnO_2_, SI-Pd/SnO_2_, and CP-Pd/SnO_2_.

The Pd content in the Pd/SnO_2_ nanoparticles estimated by WDX was 1.3 and 1.0 mol% for SI-Pd/SnO_2_ and CP-Pd/SnO_2_, respectively. This reduction in the Pd content was probably due to the weak bonding between the Pd colloidal particles or PVP and SnO_2_ surfaces. Therefore, a large portion of Pd was lost during filtration and centrifugation. Figure 4a shows the XRD patterns of the bare-SnO_2_, SI-Pd/SnO_2_, and CP-Pd/SnO_2_. The peak patterns were well-matched with the rutile-type tetragonal phase of SnO_2_ (JCPDS 41-1445), and additional peaks attributed to Pd due to low Pd concentration and other impurities were not observed. In addition, no clear shift in the SnO_2_ peaks was observed, which indicates that Pd was not incorporated into the SnO_2_ lattice. The pore diameter distributions are shown in Figure 4b. The peak pore diameters of bare-SnO_2_ and SI-Pd/SnO_2_ were the same, whereas those of CP-Pd/SnO_2_ were slightly smaller. The specific surface areas of bare-SnO_2_, SI-Pd/SnO_2_, and CP-Pd/SnO_2_ were 23.9, 24.5, and 25.3 m^2^·g^−1^, respectively. The specific surface areas were slightly increased by Pd loading. In addition, the specific surface area of CP-Pd/SnO_2_ was slightly larger than that of SI-Pd/SnO_2_, even though the Pd loading of CP-Pd/SnO_2_ was smaller. This indicates that the Pd nanoparticles of CP-Pd/SnO_2_ were smaller in size and more dispersed than those of SI-Pd/SnO_2_. The PVP protection layer may restrict the growth and aggregation of Pd nanoparticles during calcination.

**Figure 5:**
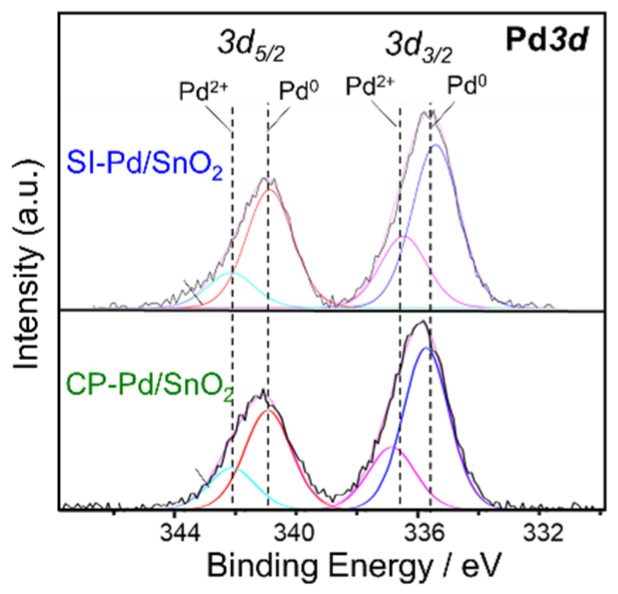
XPS spectrum of Pd*3d* using SI-Pd/SnO_2_ and CP-Pd/SnO_2_.

XPS analysis was performed to evaluate the oxidation state of the Pd on the SnO_2_ surface. The XPS spectra of Pd*3d* using SI-Pd/SnO_2_ and CP-Pd/SnO_2_ are shown in Figure 5, and the two peaks obtained consisted of Pd3*d*_5/2_ and Pd3*d*_3/2_. Based on the peak analysis, each peak can be separated into two peaks. According to the literature, Pd*3d* can be separated into two types of Pd states: Pd^0^ as a metallic state and Pd^2+^ as an oxidized state, and the peak of Pd^2+^ appears at a high binding energy [26,37]. Thus, both SI-Pd/SnO_2_ and CP-Pd/SnO_2_ have metallic Pd and PdO phases. According to the literature, the surface of Pd nanoparticles appears to be in the oxidized phase, and the inside of the Pd nanoparticles appears to be Pd [28,30]. 

**Figure 6:**
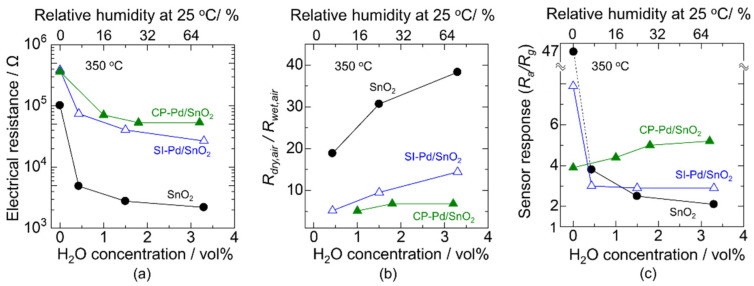
H_2_O concentration dependence of the (**a**) electrical resistance in air atmosphere, (**b**) ratio of the electrical resistance in dry air to that in wet air, and (**c**) sensor response to 200 ppm CO at 350 °C using bare-SnO_2_, SI-Pd/SnO_2_, and CP-Pd/SnO_2_.

The electrical properties of the bare-SnO_2_, SI-Pd/SnO_2_, and CP-Pd/SnO_2_ with a 30 μm sensing layer were evaluated at 350 °C under various humidity conditions. Figure 6a shows the H_2_O concentration dependence of the electrical resistance in air atmosphere. First, the electrical resistance in dry air increased with Pd loading, and the resistance was not strongly affected by the loading method. The reason for this increase in electrical resistance is the p-n junction between SnO_2_ and PdO [28,29,30]. Subsequently, the electrical resistance decreased significantly with the increasing H_2_O concentration owing to hydroxyl poisoning. To observe more closely the effect of hydroxyl poisoning, the ratio of the electrical resistance in dry air (*R_dry, air_*) to that in wet air (*R_wet, air_*) as a function of H_2_O concentration is plotted in Figure 6b. It is clear that the ratio of the electrical resistance increased with the H_2_O concentration, and the value of Pd/SnO_2_ was clearly lower than that of bare-SnO_2_. This indicates that Pd effectively protected the Pd/SnO_2_ surface from hydroxyl poisoning. Pavelko et al. reported that Pd loading on SnO_2_ showed desorption of H_2_O at less than 145 °C, and thereby almost no hydroxyl groups were formed at higher temperatures [20]. In addition, the ratio of CP-Pd/SnO_2_ was lower than that of SI-Pd/SnO_2_, especially at high H_2_O concentrations, even though the Pd loading amount was smaller and the nanostructure was comparable. Thus, the influence of the humidity change on the electrical resistance was inhibited by Pd loading via the CP method. 

Next, we demonstrate the sensor response to 200 ppm CO under various humidity conditions. Figure 6c shows the H_2_O concentration dependence of the sensor response to 200 ppm of CO at 350 °C. In a dry atmosphere, the sensor response to CO using bare-SnO_2_ was significantly higher than that of Pd/SnO_2_. The optimum temperature for the sensor response generally depends on the gas combustion activity and gas diffusivity in the sensing layer [5,38,39]. More precisely, the sensor response increases with heating because the gas combustion activity is enhanced with the temperature. However, the gas diffusion into the sensing layer tends to be disturbed if the combustion activity is very high; that is, there is a high temperature, because the combustion is complete on the surface of the sensing layer, and the byproducts are outgoing from the sensing layer. Thus, the catalytic loading shifts the optimum sensing temperature downward, and it reduces the sensor response in the high-temperature range of 350 °C. The sensor response to CO was drastically decreased by introducing H_2_O owing to hydroxyl poisoning using bare-SnO_2_. In contrast, the reduction of the sensor response by H_2_O was weakened in the case of SI-Pd/SnO_2_, and no significant change in the response was observed by the change in the H_2_O concentration over 0.5 vol%. Moreover, the sensor response using CP-Pd/SnO_2_ showed an increasing tendency over the entire H_2_O range, including under a dry atmosphere. In other words, no significant influence of hydroxyl poisoning was observed for the CP-Pd/SnO_2_ catalyst. Based on previous research, we proposed that the Pd nanoparticle size determines the humidity dependence of the CO response [34]. To be exact, the sensor response to CO using Pd/SnO_2_ with a smaller Pd size (2.3 nm) showed a decreasing tendency toward the H_2_O concentration as SI-Pd/SnO_2_ in Figure 6c, while that with a larger Pd size (5.3 nm) showed an increasing tendency toward the H_2_O concentration as CP-Pd/SnO_2_ in Figure 6c. However, this does not match the BET analysis in this work. Unfortunately, it is unclear the reason why the behaviors of the sensor response were different between Pd loading methods. The sensor response to CO using CP-Pd/SnO_2_ was higher than that using SI-Pd/SnO_2_. When the PdO surface is the active reaction site of CO, the smaller PdO size and greater dispersion cause the high sensor response to CO in a wet atmosphere because of the expanding PdO surface. In this case, hydroxyls on the SnO_2_ probably do not disturb the CO detection reaction. Hence, the sensor response to CO of CP-Pd/SnO_2_ should be higher than that of SI-Pd/SnO_2_. On the other hand, when the hydroxyls on the Pd/SnO_2_ surface determine the sensor response to CO, the SI-Pd/SnO_2_ should show a higher CO response than CP-Pd/SnO_2_, because the ratio of the electrical resistance in dry air to that in wet air of SI-Pd/SnO_2_ is greater than that of CP-Pd/SnO_2_. Thus, an increase in the sensor response to CO in a wet atmosphere using Pd/SnO_2_ was probably determined by the electronic sensitization effect of PdO on the SnO_2_ surface. Therefore, CP-Pd/SnO_2_ seems to be better for expanding the humidity resistance range while maintaining a high sensor response.

### 3.2. Improving the Sensor Response in a Humid Atmosphere

**Figure 7:**
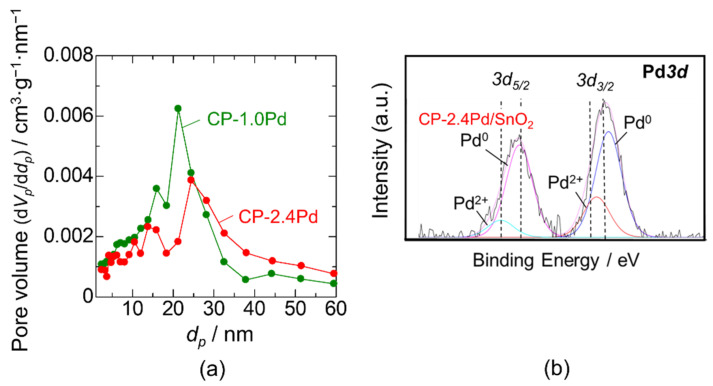
(**a**) Pore distribution of CP-1.0Pd/SnO_2_ and CP-2.4Pd/SnO_2_. (**b**) XPS spectrum of Pd*3d* using CP-2.4Pd/SnO_2_.

Collaboration between wide-range humidity resistance and a high sensor response is crucial for designing the sensor. The sensor response of CP-Pd/SnO_2_ was not sufficiently high, with a value of approximately 4–5 for the entire humidity measurement range. Thus, there remains a second question on how to improve the sensor response while maintaining humidity resistance. First, the approach of increasing the Pd loading amount was attempted, which reduced hydroxyl poisoning and enhanced CO combustion. Pd loading was carried out using the CP method, and the Pd content was increased by adjusting the total amount of the ethanol and water mixture. Even though the preparation amount of Pd acetate was the same (3 mol%), decreasing the total amount of the ethanol–water mixture to half increased the final Pd content in the Pd/SnO_2_ powder. The Pd content was estimated to be 2.4 mol% by WDX, and thereby 2.4 and 1.0 mol% CP-Pd/SnO_2_ were described as CP-2.4Pd/SnO_2_ and CP-1.0Pd/SnO_2_, respectively. Unfortunately, it is still unclear why the Pd loading amount was increased with decreasing the amount of the ethanol–water mixture. The specific surface area of CP-2.4Pd/SnO_2_ was approximately 22.4 m^2^·g^−1^, which was smaller than that of CP-1.0 Pd/SnO_2_ and bare-SnO_2_. The pore diameter distributions of both CP-Pd/SnO_2_ catalysts are shown in Figure 7a. The number of pores smaller than 25 nm clearly decreased with the increase in Pd loading. Accordingly, increasing the Pd loading probably led to the aggregation of Pd particles and filling of the small pores. The XPS spectrum of Pd*3d* using CP-2.4Pd/SnO_2_ is shown in Figure 7b. The XPS spectra of 3*d*_5/2_ and 3*d*_3/2_ were clearly separated into two peaks, which were attributed to metallic Pd (Pd^0^) and oxidized Pd (Pd^2+^). Hence, the PdO phase was also formed in CP-2.4Pd/SnO_2_ as in CP-1.0Pd/SnO_2_.

**Figure 8:**
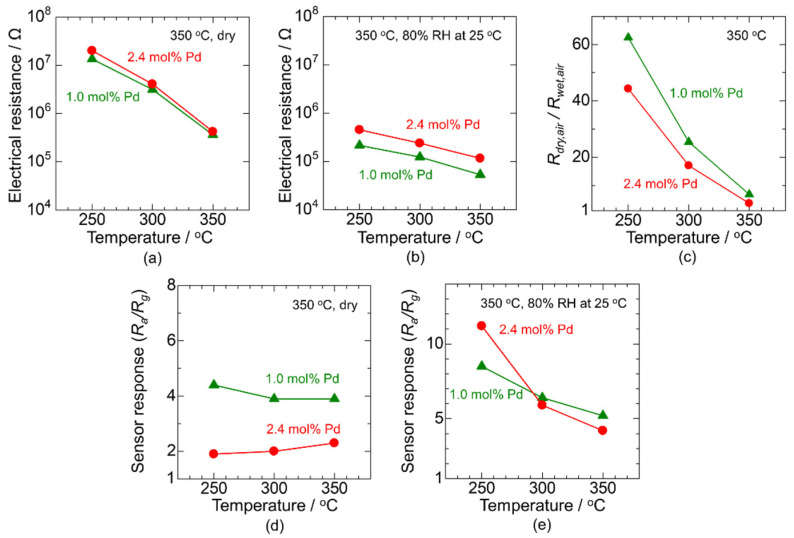
Temperature dependence of the electrical resistance in (**a**) dry air and (**b**) wet air, and (**c**) ratio of the electrical resistance in these atmospheres, and temperature dependence of the sensor response to 200 ppm CO in (**d**) dry and (**e**) wet atmospheres. The demonstration was carried out using CP-1.0Pd/SnO_2_ and CP-2.4Pd/SnO_2_. The humidity of the wet atmosphere was approximately 80% RH at 25 °C.

The electrical resistance and sensor response to 200 ppm CO were evaluated using CP-2.4Pd/SnO_2_ and CP-1.0Pd/SnO_2_ with a 30 μm sensing layer. Figure 8a,b show the temperature dependence of the electrical resistance in air under dry and wet (80% RH at 25 °C) atmospheres, respectively. As a semiconductor material, the electrical resistance increases with the decreasing temperature, and the increasing trend of the electrical resistance with cooling in wet conditions is smaller than that in dry conditions. The ratio of the electrical resistance in dry to wet conditions as a function of the operating temperature is shown in Figure 8c. The ratio increases with cooling. This is because the lower the temperature, the larger the amount of humidity adsorbed on the surface [14]. In addition, increasing the Pd loading reduced the ratio for the entire measurement temperature range. This indicates that Pd acts as a humidity-resistant additive. The sensor responses to 200 ppm CO as a function of the operating temperature in dry and wet (80% RH at 25 °C) atmospheres are shown in Figure 8d,e, respectively. In a dry atmosphere, the effect of the operating temperature on the sensor response was small for both Pd/SnO_2_ catalysts. It is likely that the high CO combustion activity of the Pd nanoparticles restricts the diffusion of CO into the sensing layer; that is, the utilization efficiency of the sensing layer is very low. In contrast, in a wet atmosphere, the sensor response clearly increased with cooling, and the positive effect of the Pd loading amount appeared only at 250 °C. The CO combustion activity of Pd is generally high at high operating temperatures, and therefore a large Pd loading amount significantly disturbs the CO diffusion into the sensing layer, even in a wet atmosphere. 

**Figure 9:**
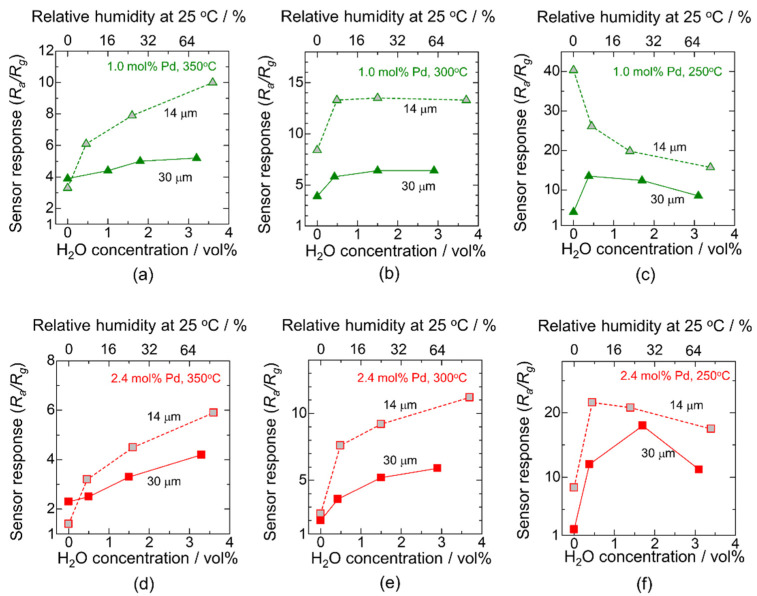
H_2_O concentration dependence of the sensor response to 200 ppm CO at (**a**,**d**) 350 °C, (**b**,**e**) 300 °C, and (**c**,**f**) 250 °C using (**a**–**c**) CP-1.0Pd/SnO_2_ and (**d**–**f**) CP-2.4Pd/SnO_2_ with different thicknesses of the sensing layer, 14 and 30 μm.

The thickness of the sensing layer is a crucial factor for improving the sensor response because of the utilization efficiency of the sensing layer [40]. Accordingly, we controlled the thickness of the sensing layer by adjusting the ratio between the Pd/SnO_2_ powder and α-terpineol. The thickness was controlled to approximately 30 μm, which is the sensor thickness discussed above, and 14 μm. Figure 9 shows the H_2_O concentration dependence of the sensor response to 200 ppm CO at 350, 300, and 250 °C using CP-1.0Pd/SnO_2_ and CP-2.4Pd/SnO_2_. It is clear that the sensor response using the 14 μm sensing layer is higher than that at 30 μm, except for the dry atmosphere at 350 °C. Thus, the utilization efficiency of the sensing layer affects the sensor response; that is, the CO combustion activity of Pd restricts the diffusion of CO into the sensing layer. According to the results, the sensor responses to CO at 300 and 350 °C as a function of H_2_O concentration exhibited an increasing tendency, regardless of the Pd loading amount and thickness of the sensing layer. The results show that strong increasing trends in the sensor response to H_2_O concentration were obtained in the thinner sensing layer. In contrast, when the temperature was lowered to 250 °C, the sensor response decreased with the increasing H_2_O concentration. This may have been due to hydroxyl poisoning. Moreover, it is noteworthy that the sensor response using CP-1.0Pd/SnO_2_ at 300 °C with a 14 μm sensing layer exhibited wide-range independence toward the change in the H_2_O concentration with a high sensor response of over 10, as shown in Figure 9b. Thus, the balance of the Pd loading method, Pd amount, and thickness of the sensing layer accomplishes the collaboration of wide-range humidity resistance and a high sensor response. According to the best of our knowledge, a lot of researchers approached to enhance the humidity resistance using catalytic additives on metal oxide surfaces such as SnO_2_ and In_2_O_3_ [24,25,26,27,41,42,43,44,45,46]. Almost all of them were focused on the metallic property using Pd, Au, Pt, their composites, and alloys, while our study only used Pd as an additive and we adjusted the characteristics of Pd by the loading method. The obtained humidity resistance of this study was comparable with the high-performance gas sensing material using a complex alloy, even though we used simple Pd/SnO_2_. Thus, designs of the Pd/SnO_2_ from the Pd loading method, Pd loading amount, operating temperature, and thickness of the sensing layer are attractive parameters to provide the humidity resistance as choosing the additives. 

## 4. Conclusions

In this study, a semiconductor-type CO gas sensor with wide-range humidity resistance and high sensitivity was developed using the Pd loading method as the material design and the thickness of the sensing layer as the sensor element design. Two types of Pd loading methods were applied, starting from Pd acetate: one was based on the SI method, and the other was based on the CP method using PVP. The specific surface area of CP-Pd/SnO_2_ with 1.0 mol% Pd was slightly larger than that of SI-Pd/SnO_2_ with 1.3 mol% Pd. According to XPS analysis, Pd nanoparticles of Pd/SnO_2_ were constructed by the oxidized PdO and metallic Pd phases. The electrical resistance demonstration showed that Pd loading restricted the significant decrease in the electrical resistance and CO sensor response by hydroxyl poisoning at 350 °C. It was probably caused by the electronic sensitization of Pd on the SnO_2_ surface. In addition, the CO response of CP-Pd/SnO_2_ was higher than that of SI-Pd/SnO_2_. Furthermore, we achieved a wide-range humidity resistance and high CO response value by thinning the sensing layer to half and lowering the sensor working temperature to 300 °C. Therefore, we can say that the balance of the Pd loading method, thickness of the sensing layer, and sensor working temperature accomplishes the collaboration of wide-range stability toward humidity change and a high sensor response. Unfortunately, we still have not determined the quantitative design of the Pd characteristics such as size, oxidation state, and dispersity on SnO_2_. We will clarify the relationship between characteristics of Pd and the loading method in the near future. Moreover, long-term stability and rapid detection ability are important factors for the practical semiconductor gas sensors, and these parameters are certainly strengthened by optimal Pd loading. Therefore, we will establish the overall design of the materials and sensing layer to develop highly stable, sensitive, and rapid detective semiconductor gas sensors.
